# High‐sensitivity C‐reactive protein is a predictor of all‐cause mortality in a rural Japanese population

**DOI:** 10.1002/jcla.25015

**Published:** 2024-02-28

**Authors:** Ryuichi Kawamoto, Asuka Kikuchi, Daisuke Niomiya, Teru Kumagi

**Affiliations:** ^1^ Department of Community Medicine Ehime University Graduate School of Medicine Toon‐City Ehime Japan; ^2^ Department of Internal Medicine Seiyo Municipal Nomura Hospital Seiyo‐City Ehime Japan

**Keywords:** all‐cause mortality, community‐dwelling persons, high‐sensitivity C‐reactive protein

## Abstract

**Background:**

High‐sensitivity C‐reactive protein (hsCRP) is a sensitive marker of inflammation. This study aimed to determine whether increased hsCRP levels are associated with all‐cause mortality rate.

**Methods:**

We examined data for participants from the 2002 Nomura Cohort Study who attended follow‐ups for 20 years (follow‐up rate: 93.3%). Of these, 793 were male (aged 61 ± 14 years) and 1040 were female (aged 63 ± 11 years). The Japanese Basic Resident Registry provided data on adjusted relative hazards for all‐cause mortality. The data were subjected to a Cox regression analysis using a time variable of age and confounding risk factors.

**Results:**

The median (interquartile range) follow‐up period was 6548 days (6094–7452 days). The follow‐up confirmed that there were 632 (34.8%) deaths, of which 319 were male (40.2% of all males) and 313 were female (30.6% of all females). Multivariable‐adjusted hazard ratio (1.27; 95% confidence interval, 1.01–1.59) in the highest hsCRP category was also significantly higher compared with reference. A higher hsCRP was associated with a greater risk of all‐cause mortality in male participants aged ≥65 years, a BMI < 25 kg/m^2^, and no history of CVD or diabetes, and this association was particularly significant among participants with both of the latter two risk factors (*p* = 0.004 and 0.022 for interaction, respectively).

**Conclusions:**

Our results indicate a significant association between hsCRP levels and all‐cause mortality in a rural Japanese population. Specifically, hsCRP appears to be a crucial biomarker for predicting long‐term survival, particularly among older persons.

## Graphical abstract

Our results indicated a significant association between hsCRP levels and all‐cause mortality in a rural Japanese population. Specifically, hsCRP appears to be a crucial biomarker for predicting long‐term survival, particularly among older persons.

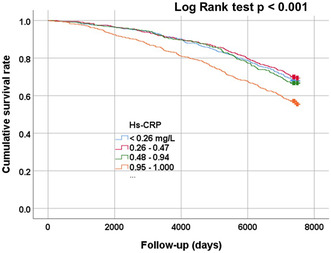

## 1 INTRODUCTION

High‐sensitivity C‐reactive protein (hsCRP) is an acutephase reactant and a highly responsive indicator of inflammation. Its primary role is to defend against bacteria and facilitate the removal of damaged cells. In healthy individuals, circulating CRP is non‐inflammatory; however, it undergoes structural changes in response to tissue damage, activating the complement system and triggering an immune response.^1^ Regulation of CRP is influenced by proinflammatory cytokines such as interleukin (IL)‐6, IL‐1, and tumor necrosis factor‐α. CRP has consequently been proven to be a valuable indicator of both infection presence and trauma severity.

Advancements in methodologies have enabled the detection of hsCRP, associated with chronic non‐communicable diseases (NCDs) characterized by an activated proinflammatory state.^1^ Numerous investigations have substantiated the notion that hsCRP, even when falling within the established clinical normal range, holds significance as an antecedent for the onset of hypertension,^2^ type 2 diabetes,^3^, ^4^, ^5^ and metabolic syndrome (MetS).^6^, ^7^ Furthermore, it might be an autonomous prognostic factor indicative of the initial phases of cardiovascular disease (CVD).^8^, ^9^, ^10^, ^11^ In most instances, it is related to the primary ‘conventional’ risk factors, including gender, age, tobacco use, cholesterol levels, blood pressure, and diabetes.^12^ Moreover, many research studies have explored the links between hsCRP levels and mortality rates. For example, a recent study demonstrated that heightened hsCRP levels are connected to an increased risk of overall mortality.^8^, ^9^, ^13^, ^14^, ^15^, ^16^, ^17^, ^18^, ^19^, ^20^ Nevertheless, many of these studies are clinical trials or hospital‐based studies and are unlikely to reflect the basic health status of the general population. In addition, in these studies, low‐grade systemic inflammation can be defined by hsCRP levels <1.00, 1.00–3.00, and >3.00 mg/L indicating lower, average, or higher relative cardiovascular risk, respectively.^21^ Japanese have lower levels of CRP than their Western counterparts, in part due to lower levels of adiposity and lower body mass index (BMI), and the hsCRP cut‐off point for high‐risk of future development of CHD is likely to be >1.00 mg/L.^22^ To date, few studies have examined the association between hsCRP levels and all‐cause mortality in a Japanese population.^17^

This study sought to investigate the potential link between elevated hsCRP levels and all‐cause mortality while also assessing the independence of this association to gender, BMI, and other factors that could confound CVD outcomes. We conducted an analysis using cohort data from Japanese community‐dwelling individuals.

## 2 MATERIALS AND METHODS

### 2.1 Subjects

**FIGURE 1:**
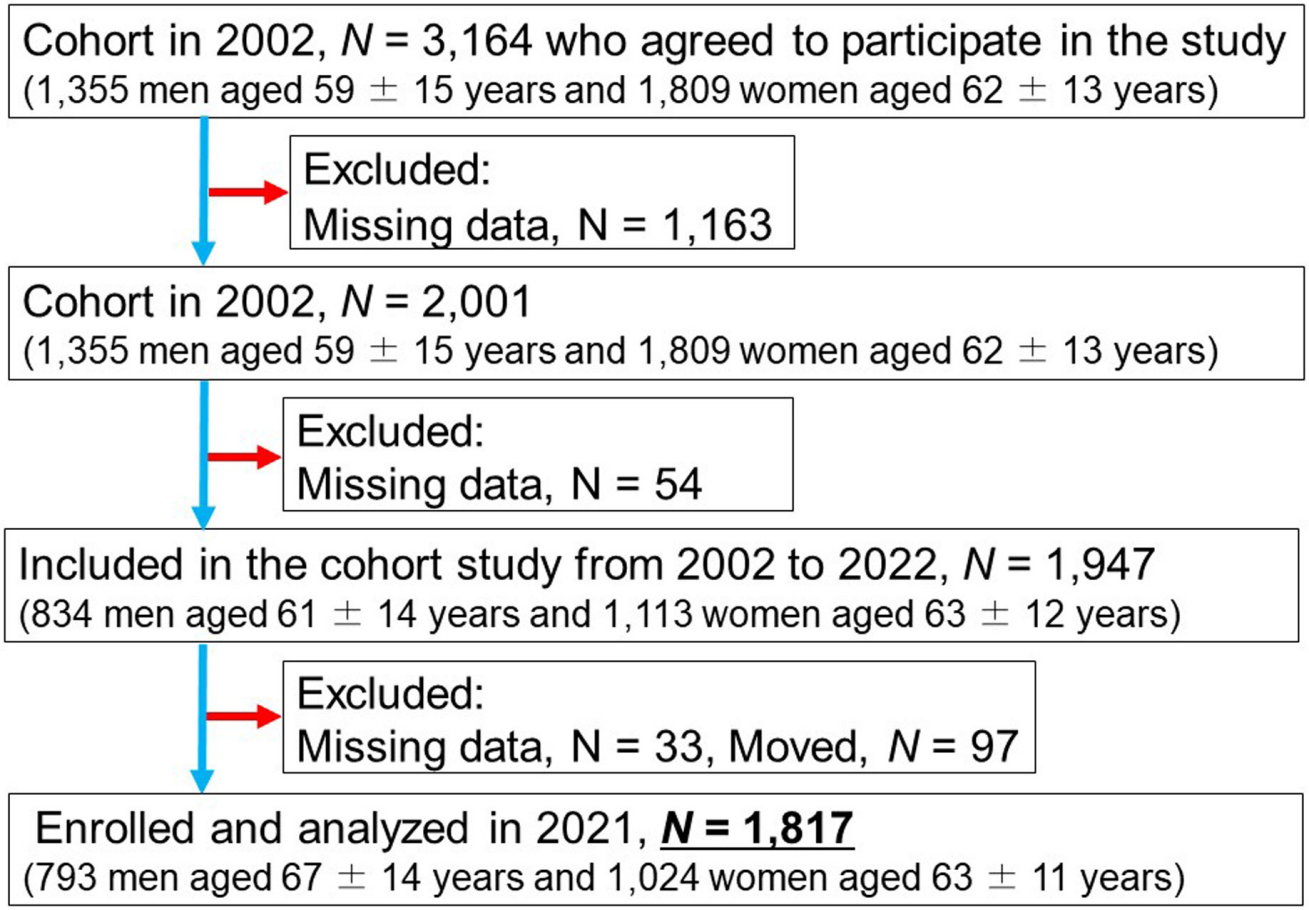
Participant flowchart.

The investigation was initiated in 2002 and focused on residents of the local area. Participants mainly included individuals from rural Ehime Prefecture and those who underwent annual community‐based health examinations. The study included 3164 individuals who were between 20 and 89 years of age at the time of enrollment and who had undergone a yearly physical examination for community residents. Each participant completed a self‐administered survey encompassing inquiries about physical activity, medical background, current health status, and usage of prescribed medications such as antihypertensive, antidyslipidemic, and antidiabetic drugs. The participant selection and exclusion processes are depicted in Figure 1. This group conducted an initial assessment, after which 2001 individuals provided an overnight fasting plasma sample (>11 h) for hsCRP. Subsequently, 1817 participants were followed for 20 years, and the Basic Resident Ledger database, which records information on Japanese citizens, was utilized to confirm their survival and mortality status (survival or deceased). All protocols were approved by the Ethics Committee of Ehime University School of Medicine (approval no. 1903018), and each participant provided informed consent before participating.

### 2.2 Evaluation of risk factors

Data on demographic characteristics and risk factors were procured from clinical records. BMI was computed by dividing weight (in kilograms) by the square of height (in meters). Blood pressure readings were taken with a suitable cuff on the upper arm's right side while participants were seated in a relaxed position following a minimum of 5 min of rest. This was done using an automatic oscillometric blood pressure recorder (BP‐103i; Colin, Aichi, Japan). Smoking categories were nonsmokers, ex‐smokers, light smokers (<20 pack‐years), and heavy smokers (≥20 pack‐years). The quantity of alcohol consumed daily was gauged using the Japanese liquor unit, where each unit corresponds to 22.9 g of ethanol. Participants were grouped as nondrinkers, occasional drinkers (<1 unit/day), light daily drinkers (1–2 units/day), and heavy daily drinkers (2–3 units/day). No participants consumed more than 3 units/day. Measurements for triglycerides (TG), high‐density lipoprotein cholesterol (HDL‐C), low‐density lipoprotein cholesterol (LDL‐C), blood glucose (BG), creatinine (Cr), and hsCRP level were taken during fasting. Serum hsCRP level was measured through a Behring BN II nephelometer (Dade Behring Inc., Marburg, Germany), with inter‐ and intra‐assay coefficients of variation at 3.2% and 6.7%, respectively. The Japanese‐adjusted coefficients were integrated into the chronic kidney disease (CKD) epidemiology equation (CKD‐EPI) for calculating the estimated glomerular filtration rate (eGFR). In males with a serum Cr level of 0.9 mg/dL or lower, the equation took the form of 141 × (Cr/0.9)^−0.411^ × 0.993^(age)^ × 0.813. For cases where the Cr level exceeded 0.9 mg/dL, the equation was altered to 141 × (Cr/0.9)^−1.209^ × 0.993^(age)^ × 0.813. In parallel, for females with a Cr level of 0.7 mg/dL or less, the equation was 144 × (Cr/0.7)^−0.329^ × 0.993^(age)^ × 0.813. When the Cr level surpassed 0.7 mg/dL, the equation transformed to 144 × (Cr/0.7)^−1.209^ × 0.993^(age)^ × 0.813.^23^

### 2.3 Statistical analysis

Statistical analysis was performed using SPSS Statistics 27.0 (SPSS, Chicago, IL, USA) for statistical data evaluation. Continuous variables are presented as mean ± standard deviation (SD). Variables not following a normal distribution (e.g., TG, BG, and hsCRP) are represented by their median and interquartile range (IQR). Log‐transformed values were applied for parameters exhibiting non‐normal distributions in all analyses. Participants were classified into four categories as 1st quartile (<0.26 mg/L), 2nd quartile (0.26–0.47 mg/L), 3rd quartile (0.48–0.94 mg/L), or 4th quartile (>0.95 mg/L), according to the quartile of their hsCRP levels. Categorical variables were compared using chi‐squared tests, while analysis of variance (ANOVA) was employed for normally distributed continuous variables. Spearman's correlations (rho) were computed to reveal multilinear relationships among various features. Each baseline characteristic underwent univariate analysis using a Cox proportional hazards model, with significant confounding factors included as covariates. Following this, a multivariable analysis was conducted in the same model framework utilizing a forced‐entry approach, with age as the primary time variable. Sensitivity analyses were conducted to assess the consistency of the observed relationship between hsCRP levels and all‐cause mortality. In addition, we conducted an additional analysis excluding individuals who died within less than 3 years of the study to eliminate the impact of pre‐existing conditions (such as cancer) that subjects originally had. Likelihood ratio tests were employed to scrutinize interactions between hsCRP groups and subgroup variables. An interaction test was executed to evaluate the effect variable, with adjustments made for all significant confounding variables (except the effect variable). All reported *p*‐values were two‐sided, and statistical significance was set at *p* < 0.05.

## 3 RESULTS

### 3.1 Background characteristics

The study included 793 men, aged 61 ± 14 (range, 20–89) years, and 1024 women, aged 63 ± 11 (range, 22–88) years. The characteristics of participants in relation to the baseline hsCPR category are illustrated in Table 1. The median hsCRP level was 0.47 (IQR: 0.25–0.94) mg/L. Older participants with a higher BMI were more likely to report a higher hsCRP. Participants in higher hsCRP category quartiles were more likely to exhibit an increased prevalence of male gender, smoking habits, CVD, hypertension, hypertriglyceridemia, hypo‐HDL cholesterolemia, hyper‐LDL cholesterolemia, diabetes, and CKD. The prevalence of use of lipid‐lowering medication showed no significant difference.

**TABLE 1:** Participant baseline characteristics stratified by high‐sensitivity C‐reactive protein category.

Baseline characteristics	Baseline hsCRP categories (mg/L)					*p* for trend
	Total *N* = 1817	<0.26 *n* = 467	0.26–0.47 *n* = 449	0.48–0.94 *n* = 448	0.95–10.0 *n* = 453	
Gender (male), *n* (%)	793 (43.6)	163 (34.9)	193 (43.0)	212 (47.3)	225 (49.7)	**<0.001**
Age (years)	62 ± 12	60 ± 13	61 ± 12	63 ± 12	64 ± 12	**<0.001**
Body mass index (kg/m^2^)	23.5 ± 3.2	22.0 ± 2.8	23.3 ± 2.9	24.0 ± 3.1	24.8 ± 3.5	**<0.001**
Smoking habits (never/past/light/heavy), %	73.4/11.2/6.9/8.6	79.9/7.3/7.3/5.6	73.3/11.8/7.8/7.1	72.3/12.7/4.9/10.0	67.8/13.0/7.5/11.7	**0.001**
Drinking habits (never/occasional/light/heavy), %	42.5/29.3/17.9/10.2	42.8/33.2/14.6/9.4	42.3/28.5/21.6/7.6	42.6/26.6/17.6/13.2	42.4/28.9/18.1/10.6	**0.044**
History of cardiovascular disease, *n* (%)	147 (8.1)	32 (6.9)	24 (5.3)	37 (8.3)	54 (11.9)	**0.002**
Hypertension, *n* (%)	1012 (55.7)	231 (49.5)	235 (52.3)	267 (59.6)	279 (61.6)	**<0.001**
Systolic blood pressure (mmHg)	139 ± 22	136 ± 22	138 ± 22	142 ± 21	141 ± 21	**<0.001**
Diastolic blood pressure (mmHg)	82 ± 12	80 ± 12	82 ± 12	83 ± 11	83 ± 11	**0.001**
Use of antihypertensive medication, *n* (%)	463 (25.5)	8.2 (17.6)	104 (23.2)	128 (28.6)	149 (32.6)	**<0.001**
Hypertriglyceridemia, *n* (%)	330 (18.2)	57 (12.2)	76 (16.9)	92 (20.5)	105 (23.2)	**<0.001**
Triglycerides (mg/dL)	93 (70–130)	84 (62–115)	93 (71–127)	94 (72–136)	100 (72–144)	**<0.001**
Hypo‐HDL cholesterolemia, *n* (%)	96 (5.3)	13 (2.8)	15 (3.3)	25 (5.6)	43 (9.5)	**<0.001**
HDL cholesterol (mg/dL)	62 ± 16	66 ± 16	64 ± 15	61 ± 15	58 ± 15	**<0.001**
Hyper‐LDL cholesterolemia, *n* (%)	522 (28.7)	110 (23.6)	128 (28.5)	133 (29.7)	151 (33.3)	**0.012**
LDL cholesterol (mg/dL)	119 ± 31	115 ± 29	119 ± 30	122 ± 33	121 ± 33	**0.004**
Use of lipid‐lowering medication, *n* (%)	99 (5.4)	15 (3.2)	28 (6.2)	24 (5.4)	32 (7.1)	0.061
Diabetes, *n* (%)	132 (7.3)	18 (3.9)	26 (5.8)	37 (8.3)	51 (11.3)	**<0.001**
Blood glucose (mg/dL)	94 (88–101)	92 (86–98)	93 (88–100)	95 (89–103)	95 (89–105)	**<0.001**
Use of anti‐diabetic medication, *n* (%)	66 (3.6)	14 (3.0)	12 (2.7)	16 (3.6)	24 (5.3)	**0.150**
Chronic kidney disease, *n* (%)	160 (8.8)	30 (6.4)	32 (7.1)	39 (8.7)	59 (13.0)	**0.002**
eGFR (mL/min/1.73 m^2^)	80.4 ± 17.3	81.8 ± 16.6	81.2 ± 16.9	80.5 ± 17.1	78.1 ± 18.3	**0.006**

*Note*: Data are presented as mean ± SD. Data for triglycerides and blood glucose were skewed and are thus presented as median (interquartile range) values and log‐transformed for analysis.* p* *‐*values are from ANOVA tests for continuous variables or χ^2^ tests for categorical variables. Significant values (*p* < 0.05) are presented in bold.

Abbreviations: eGFR, estimated glomerular filtration ration; HDL, high‐density lipoprotein; hs‐CRP, high‐sensitivity C‐reactive protein; LDL, low‐density lipoprotein.

### 3.2 All‐cause mortality and mortality rate of participants stratified by baseline hsCRP

**FIGURE 2:**
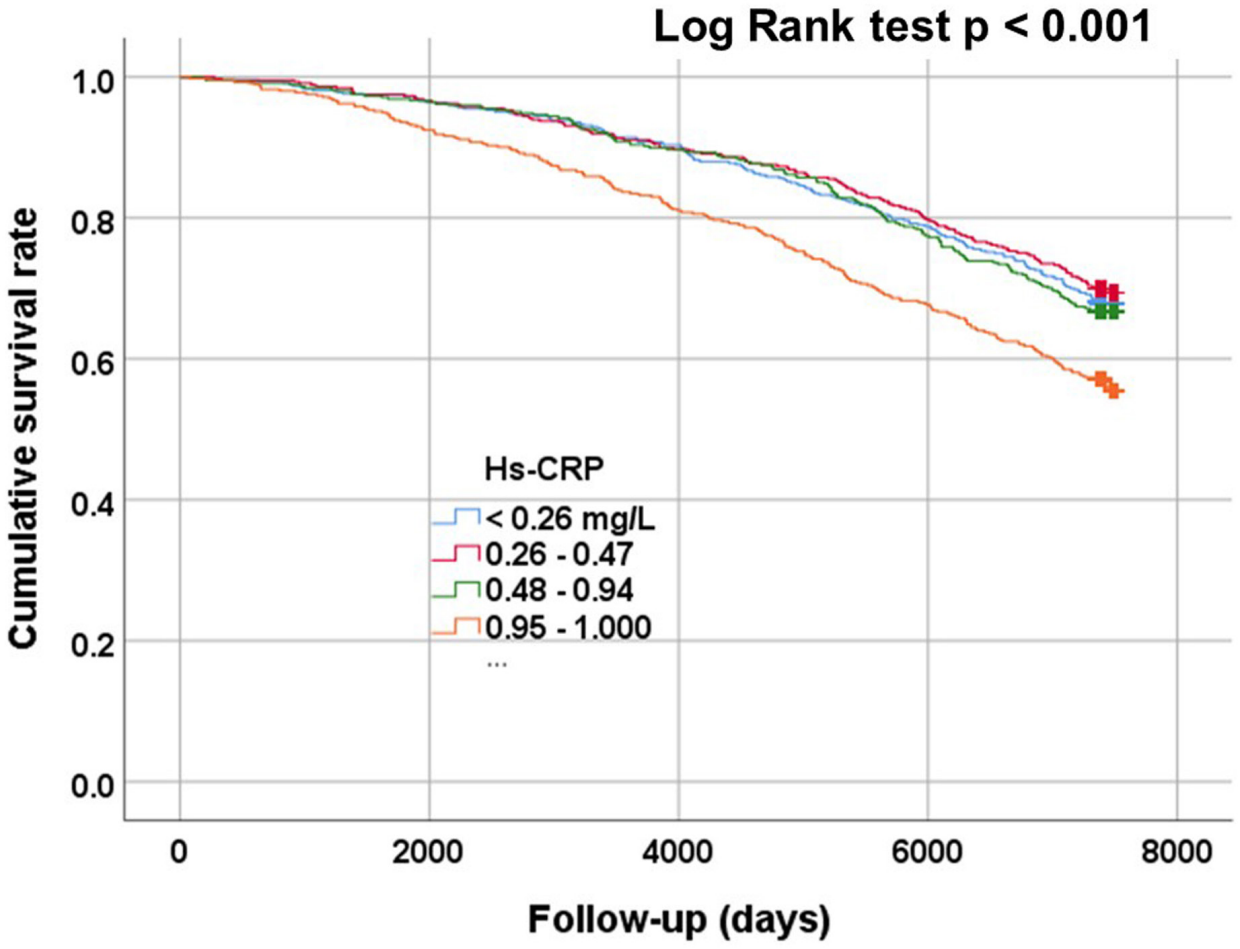
Analysis of the association between high‐sensitivity C‐reactive protein (hsCRP) categories and all‐cause mortality during follow‐up. Participants were classified into four categories as 1st quartile (<0.26 mg/L), 2nd quartile (0.26–0.47 mg/L), 3rd quartile (0.48–0.94 mg/L), or 4th quartile (>0.95 mg/L) categories, according to the standard deviation of their hsCRP levels. Higher hsCRP levels were significantly associated with lower cumulative survival rate (log‐rank test: *p* < 0.001).

During a median (interquartile range) follow‐up period of 6548 days (6094–7452 days), a total of 632 (34.8%) all‐cause deaths occurred (men: 319 [40.2%] and women: 313 [30.6%]). Table 2 displays the number of all‐cause deaths and mortality rates at 5, 10, and 20 years categorized by baseline hsCRP quartile. In all instances, the mortality rate in the fourth hsCRP quartile was significantly higher. Figure 2 shows Kaplan–Meier survival curves for cumulative survival rates to identify patterns in the relationships between the hsCRP quartiles and all‐cause mortality. The results indicate that the fourth hsCRP quartile (0.95–10.0 mg/L) has the lowest cumulative survival rate among quartiles for participants (log‐rank test: *p* < 0.001).

**TABLE 2:** All‐cause mortality rate of participants stratified by high‐sensitivity C‐reactive protein category.

All‐cause mortality, *n* (%)	Baseline hsCRP categories (mg/L)					*p* for trend
	Total *N* = 1817	<0.26 *n* = 467	0.26–0.47 *n* = 449	0.48–0.94 *n* = 448	0.95–10.0 *n* = 453	
5 years later	69 (3.8)	13 (2.8)	12 (2.7)	14 (3.1)	30 (6.6)	**0.004**
10 years later	198 (19.9)	40 (8.6)	40 (8.9)	43 (9.6)	75 (16.6)	**<0.001**
20 years	632 (34.8)	150 (32.1)	136 (30.3)	149 (33.3)	197 (43.5)	**<0.001**

*Note*: *p‐*values are from χ^2^ tests for categorical variables. Significant values (*p* < 0.05) are presented in bold.

### 3.3 Hazard ratios and 95% confidence intervals of baseline hsCRP for all‐cause mortality

Table S1 shows Spearman's correlations (rho) among the various confounders. No strong correlations were observed between the different factors that would lead to multicollinear correlations. Table 3 presents the HRs and 95% confidence intervals (CIs) for the baseline hsCRP (continuous data) for all‐cause mortality. The HRs for all‐cause mortality were significantly higher in participants with a higher hsCRP as well as male gender, aging, a smaller BMI, the presence of hypertension, diabetes, and CKD (*p* < 0.001).

**TABLE 3:** Hazard ratios and 95% confidence intervals of baseline characteristics for all‐cause mortality.

Baseline characteristics *n* = 1817	Non‐adjusted HR (95% CI)	Adjusted HR (95% CI)
Gender (male = 1, female = 2), per 1	**0.70 (0.60–0.82)**	**0.71 (0.57–0.89)**
Age (years), per 1	**1.13 (1.12–1.14)**	**1.12 (1.11–1.13)**
Body mass index, per 1	**0.96 (0.93–0.98)**	**0.96 (0.93–0.99)**
Smoking habits (never = 1/past = 2/light = 3/heavy = 4), per 1	1.06 (0.94–1.10)	**1.13 (1.02–1.25)**
Drinking habits (never = 1/occasional = 2 L/light =3/heavy = 4), per 1	0.94 (0.87–1.02)	0.98 (0.88–1.09)
History of cardiovascular disease (no = 1, yes = 2), per 1	3.19 (0.58–3.94)	**1.48 (1.19–1.85)**
Hypertension (no = 1, yes = 1), per 1	**2.53 (2.13–3.02)**	1.10 (0.91–1.33)
Hypertriglyceridemia (no = 1, yes = 2), per 1	0.89 (0.73–1.10)	0.99 (0.79–1.24)
Low HDL‐cholesterolemia (no = 1, yes = 2), per 1	1.04 (0.74–1.46)	0.80 (0.55–1.15)
Hyper LDL cholesterolemia (no = 1, yes = 2), per 1	1.02 (0.86–1.21)	0.93 (0.78–1.11)
Diabetes (no = 1, yes = 2), per 1	**1.64 (1.27–2.13)**	**1.55 (1.18–2.02)**
Chronic kidney disease (no = 1, yes = 2), per 1	**2.52 (2.04–3.12)**	1.11 (0.88–1.38)
Hs‐CRP category, per 1	**1.16 (1.08–1.24)**	**1.28 (1.08–1.52)**

*Note*: Data for hsCRP were skewed and log‐transformed for analysis. Significant values (*p* < 0.05) are presented in bold.

Abbreviations: CI, confidence interval; HDL, high‐density lipoprotein; HR, hazard ratio; hsCRP, high‐sensitivity c‐reactive protein; LDL, low‐density lipoprotein.

Table 4 indicates that participants in the highest hsCRP category (0.95–10.0 mg/L; model 1: HR, 1.52; 95% CI, 1.23–1.88) had a higher risk of all‐cause mortality than those in the reference category (<0.26 mg/L). This analysis was adjusted for age, gender, BMI, smoking status, drinking habits, history of CVD, hypertriglyceridemia, hypo‐HDL cholesterolemia, hyper‐LDL cholesterolemia, diabetes, and CKD. In each model, multivariable‐adjusted HR (model 4: HR, 1.27; 95%CI, 1.01–1.59) in the highest hsCRP category was also significantly higher compared with reference.

**TABLE 4:** Hazard ratios and 95% confidence intervals of baseline high‐sensitivity C‐reactive protein categories for all‐cause mortality.

*n* = 1817	Non‐adjusted and adjusted HR (95% CI) Baseline hsCRP categories (mg/L)				
	<0.26 *n* = 467	0.26–0.47 *n* = 449	0.48–0.94 *n* = 448	0.95–1.00 *n* = 453	*p* for trend
Prevalence of death (%)	150 (32.1)	136 (30.3)	149 (33.3)	197 (43.5)	**<0.001**
Model 1	Reference	0.93 (0.74–1.18)	1.04 (0.83–1.31)	**1.52 (1.23–1.88)**	**<0.001**
Model 2	Reference	0.88 (0.69–1.10)	0.91 (0.73–1.14)	**1.26 (1.02–1.56)**	**0.003**
Model 3	Reference	0.90 (0.72–1.14)	0.96 (0.76–1.22)	**1.31 (1.05–1.64)**	**0.004**
Model 4	Reference	0.91 (0.72–1.15)	0.94 (0.75–1.19)	**1.27 (1.01–1.59)**	**0.011**

*Note*: Model 1 was non‐adjusted; Model 2 was adjusted for age and gender; Model 3 was adjusted for body mass index, smoking status, drinking habits, and history of cardiovascular disease in addition to covariates in model 2; Model 4 was adjusted for hypertriglyceridemia, hypo‐HDL‐cholesterolemia, hyper‐LDL‐cholesterolemia, diabetes, and chronic kidney disease, in addition to covariates in model 3. Significant values (*p* < 0.05) are presented in bold.

Abbreviations: CI, confidence interval; HR, hazard ratio; hsCRP, high‐sensitivity c‐reactive protein.

### 3.4 Hazard ratios and 95% confidence intervals for baseline hsCRP for all‐cause mortality per sub‐analyses

In the sensitivity analysis, Table 5 shows stratification of participants based on gender, age (< and ≥65 years), BMI (< and ≥25 kg/m^2^), smoking status (non‐current and current smoker), history of CVD (absence and presence), and diabetes (absence and presence). A higher hsCRP was associated with a greater risk of all‐cause mortality in participants who were male, aged ≥65 years, BMI < 25 kg/m^2^, and no history of CVD or diabetes, and this association was particularly significant among participants with BMI of <25 kg/m^2^ (*p* = 0.004 for interaction) and without a history of diabetes (*p* = 0.022 for interaction). Furthermore, the notable graded relationship endures even when excluding fatalities within the initial 3 years (1095 days) of the follow‐up period.

**TABLE 5:** Hazard ratios and 95% confidence intervals of baseline high‐sensitivity C‐reactive protein (continuous data) for all‐cause mortality by sub‐analysis.

Baseline characteristics *n* = 1817	Multivariable‐adjusted HR (95% CI)	*p‐*value	*p* for interaction
Gender			
Men (*n* = 793)	**1.33 (1.05–1.69)**	**0.019**	0.888
Women (*n* = 1024)	1.21 (0.94–1.55)	0.136	
Age			
<65 years (*n* = 905)	1.17 (0.74–1.83)	0.509	0.342
≥65 years (*n* = 912)	**1.27 (0.05–1.52)**	**0.012**	
Body mass index			
<25 kg/m^2^ (*n* = 1276)	**1.36 (1.12–1.65)**	**0.002**	**0.004**
≥25 kg/m^2^ (*n* = 541)	1.04 (0.73–1.48)	0.815	
Smoking status			
Non‐current smoker (*n* = 1536)	**1.23 (1.02–1.47)**	**0.027**	0.369
Current smoker (*n* = 281)	**1.84 (1.12–3.02)**	**0.015**	
History of cardiovascular disease			
Absence (*n* = 1670)	**1.28 (1.06–1.54)**	**0.012**	0.610
Presence (*n* = 147)	1.31 (0.89–1.93)	0.167	
Diabetes			
Absence (*n* = 1685)	**1.41 (1.18–1.68)**	**<0.001**	**0.022**
Presence (*n* = 132)	0.54 (0.29–1.02)	0.056	
Time to death			
<1095 days (*n* = 33)	Not examined	**0.004**	–
≥1095 days (*n* = 1784)	**1.29 (1.08–1.54)**		

*Note*: Multivariate‐adjusted HR: adjusted for age, sex, body mass index, smoking status, drinking habits, history of cardiovascular disease, hypertriglyceridemia, hypo‐HDL‐cholesterolemia, hyper‐LDL‐cholesterolemia, diabetes, and chronic kidney disease. Significant values (*p* < 0.05) are presented in bold.

Abbreviations: CI, confidence interval; HR, hazard ratio; hs‐CRP, high sensitivity c‐reactive protein.

## 4 DISCUSSION

In this study, we investigated whether hsCRP could function as a predictive factor for all‐cause mortality in a prospective study involving middle‐aged individuals from Japan at baseline and a median follow‐up of 17.69 years (32,148 person‐years). Our findings reveal a clear trend of risk of all‐cause mortality increasing with hsCRP levels. Our study outcomes validate a connection between hsCRP and all‐cause mortality and underscore its independence from potential confounding factors related to inflammation or pre‐existing chronic illnesses. Moreover, the significant graded association persists even after excluding deaths within 3 years of follow‐up, minimizing the possibility of reverse causality. Additionally, the HR remained consistent when excluding participants who self‐reported a medical history of CVD and diabetes.

Several previous cohorts show that hsCRP is an independent confounding factor for all‐cause mortality. Our results align with prior research showing significant positive links between hsCRP and all‐cause mortality, particularly in cases where hsCRP levels are higher.^8^, ^9^, ^24^, ^25^, ^26^ HsCRP > 10.0 mg/L was a stronger predictor of clinical events than a conventional cut point of 3.0 mg/L.^9^, ^25^ In this group, the hsCRP level exhibited a dose‐dependent relationship with the risk of all‐cause mortality, although the hsCRP levels were higher than in the Asian population. Analyzing the baseline data from 2008 to 2010 in a cohort of 14,238 participants in the Brazilian Longitudinal Study of Adult Health, it was observed that the risk of mortality increased progressively across quartiles of hsCRP. This increase was evident with a HR of 1.45 (95% CI, 1.05–2.01) in quartile 2 (0.70–1.39 mg/day), rising to 1.95 (95% CI, 1.42–2.69) in quartile 4 (3.02–10.0 mg/day) when compared to quartile 1 (0.09–0.70 mg/L).^18^ In a study conducted in Korea with a total of 41,070 men and 81,011 women aged 40 years or older (follow‐up duration: 6.8 years), it was found that there was a dose–response relationship between elevated hsCRP levels and the risk of all‐cause mortality in both genders.^20^ This study included 2206 participants who were 80 years of age or older (with a median age of 93.0 years) from the Healthy Aging and Biomarkers Cohort Study (follow‐up: 3.1 years). In comparison to the lowest quartile (hsCRP <0.46 mg/L), the fully adjusted HRs for the second (0.47–1.13 mg/L), third (1.14–2.92 mg/L), and fourth quartiles (2.93–10.0 mg/day) were 1.17 (95% CI, 0.94–1.46), 1.28 (95% CI, 1.01–1.61), and 1.49 (95% CI, 1.20–1.87), respectively.^19^ Furthermore, it should be noted that the association between hsCRP levels and all‐cause mortality was influenced by smoking status (*p* for interaction = 0.011), and the association of hsCRP with all‐cause mortality was modified by smoking status (*p* for interaction = 0.011). In this study, the risk of all‐cause mortality was higher among participants with hsCRP ≥0.95 mg/L, which is lower (approximately 1/10) than in Westerners, consistent with results from other Asian studies.

Although most individuals with hyperinsulinemia and chronic inflammation tend to exhibit higher BMI levels, Wiebe et al.^27^ showed that a distinct subgroup of individuals with lower BMI values also experienced hyperinsulinemia and chronic inflammation and that this subset of participants was associated with the highest mortality risk. In our study, an association between hsCRP and all‐cause mortality was significantly high in participants with a BMI of <25 kg/m^2^. Previous experimental and clinical investigations have consistently shown a notable correlation between increased CRP levels and elevated susceptibility to developing conditions such as ischemic heart disease, type 2 diabetes, neurodegenerative disorders such as Parkinson's disease and Alzheimer's disease, and both hemorrhagic and ischemic strokes. Furthermore, autoimmune diseases such as systemic sclerosis and rheumatoid arthritis have also been linked to increased CRP levels.^28^ Considering that hsCRP indicates the presence of these diseases, it is plausible that the observed relationship with mortality is more pronounced in individuals who are not subject to these underlying conditions.

Extensive research has been conducted on the relationship between hsCRP levels and all‐cause mortality. While the exact mechanisms are not fully understood, several candidate pathways have been proposed.^28^ Elevated hsCRP levels indicate chronic low‐grade inflammation in the body, which has been linked to a wide range of chronic diseases, including CVD, diabetes, cancer, and neurodegenerative conditions. These chronic diseases are the leading causes of mortality. Chronic inflammation can impair the function of the endothelium, the inner lining of blood vessels.^29^ Endothelial dysfunction can lead to vasoconstriction, reduced nitric oxide production, and increased susceptibility to blood clot formation. Chronic inflammation is also associated with insulin resistance and metabolic dysregulation.^30^

While the present study provides valuable insights into the medical situation of Japan's rural population, it was subject to some limitations. We employed a cohort approach, assessing baseline characteristics and hsCRP levels during the initial visit. However, it is important to recognize that hsCRP levels and certain covariates can fluctuate over time, potentially changing during an extended follow‐up period. Consequently, the significance of the study's findings might be underestimated rather than overestimated due to nondiscriminatory misclassification bias. Secondly, our study depended on recording all deaths, regardless of their cause, through Japan's Basic Resident Register. This approach might have excluded individuals who emigrated during the survey period. Thirdly, the baseline assessment considered numerous confounding factors, including medications, underlying diseases (e.g., cancer), and lifestyle modifications, all previously linked to mortality. Despite our efforts to account for these confounding factors through baseline physical examinations, there may still be unmeasured variables that remained unexamined in this study (e.g., diet, body shape and physique, cancer, traffic accidents, daily life accidents, or natural disasters). Therefore, further research is necessary to explore the impact of these unexamined factors. Lastly, due to the relatively small number of participants and deaths in our study, the causal relationship between hsCRP levels and all‐cause mortality may have been underestimated.

## 5 CONCLUSIONS

Current research demonstrates a robust connection between hsCRP levels and all‐cause mortality in the general population. The precise mechanism underlying this association remains unclear. However, it appears to be unrelated to conventional cardiovascular risk factors, including age, BMI, smoking habits, alcohol consumption, blood pressure, diabetes, and lipid levels. Consequently, hsCRP levels, particularly in individuals with a BMI less than 25 kg/m^2^ and without diabetes, could serve as a crucial indicator for assessing atherogenic risk and a potential target for modifying atherogenic risks

## AUTHOR CONTRIBUTIONS

RK and DN conceived the idea of the study. RK developed the statistical analysis plan and conducted statistical analyses. RK and AK contributed to the interpretation of the results. RK drafted the original manuscript and supervised the conduct of this study. All authors reviewed and revised the manuscript draft and approved the final version for publication.

## FUNDING INFORMATION

This work was partially supported by a grant‐in‐aid for scientific research from the Foundation for Development of the Community (2023). No additional external funding was received. The funders played no role in the study design, data collection and analysis, decision to publish, or manuscript preparation.

## CONFLICT OF INTEREST STATEMENT

The authors declare that they do not have any conflicts of interest.

## Supporting information


Table S1.

## Data Availability

The data that supports the findings of this study are available on request from the corresponding author. The data is not publicly available due to privacy or ethical restrictions.
