# Supplementary material for: High‐sensitivity C‐reactive protein is a predictor of all‐cause mortality in a rural Japanese population
Source: J Clin Lab Anal. 2024 Feb 28;38(4):e25015. doi: 10.1002/jcla.25015 (PMC10943256; doi:10.1002/jcla.25015)
Supplement: Supplementary file 1 — Table S1. [file JCLA-38-e25015-s001.docx]

Supplemental Table Spearman's correlations (rho) among the various confounders

|  |  | hsCRP category | Gender | Age | BMI | Smoking status | Drinking status | History of CVD | Hypertension | Hypertriglyceridemia | Low HDL-cholesterolemia | Hyper LDL cholesterolemia | Diabetes | CKDepi |
| --- | --- | --- | --- | --- | --- | --- | --- | --- | --- | --- | --- | --- | --- | --- |
| hsCRP category | Spearman’s rho | 1.000 | -0.111 | 0.100 | 0.310 | 0.096 | 0.022 | 0.074 | 0.099 | 0.107 | 0.112 | 0.076 | 0.107 | 0.084 |
|  | p-value |  | 0.000 | 0.000 | 0.000 | 0.000 | 0.342 | 0.002 | 0.000 | 0.000 | 0.000 | 0.001 | 0.000 | 0.000 |
| Gender  (male = 1, female = 2) | Spearman’s rho | -0.111 | 1.000 | 0.051 | -0.038 | -0.620 | -0.615 | -0.048 | -0.041 | -0.092 | -0.115 | 0.149 | -0.070 | -0.028 |
|  | p-value | 0.000 | . | 0.031 | 0.104 | 0.000 | 0.000 | 0.040 | 0.081 | 0.000 | 0.000 | 0.000 | 0.003 | 0.232 |
| Age | Spearman’s rho | 0.100 | 0.051 | 1.000 | -0.042 | -0.168 | -0.213 | 0.222 | 0.421 | -0.032 | -0.003 | 0.093 | 0.055 | 0.237 |
|  | p-value | 0.000 | 0.031 | . | 0.073 | 0.000 | 0.000 | 0.000 | 0.000 | 0.175 | 0.902 | 0.000 | 0.020 | 0.000 |
| Body mass index | Spearman’s rho | 0.310 | -0.038 | -0.042 | 1.000 | -0.023 | 0.015 | 0.004 | 0.202 | 0.186 | 0.123 | 0.136 | 0.088 | 0.060 |
|  | p-value | 0.000 | 0.104 | 0.073 | . | 0.337 | 0.521 | 0.872 | 0.000 | 0.000 | 0.000 | 0.000 | 0.000 | 0.010 |
| Smoking status  (never = 1/past = 2/light = 3 /heavy = 4) | Spearman’s rho | 0.096 | -0.620 | -0.168 | -0.023 | 1.000 | 0.429 | 0.025 | -0.075 | 0.102 | 0.096 | -0.105 | -0.005 | -0.019 |
|  | p-value | 0.000 | 0.000 | 0.000 | 0.337 | . | 0.000 | 0.278 | 0.001 | 0.000 | 0.000 | 0.000 | 0.839 | 0.416 |
| Drinking status  (never = 1/occasional = 2 l/light = 3/heavy = 4) | Spearman’s rho | 0.022 | -0.615 | -0.213 | 0.015 | 0.429 | 1.000 | -0.052 | -0.041 | 0.026 | -0.002 | -0.172 | -0.012 | -0.044 |
|  | p-value | 0.342 | 0.000 | 0.000 | 0.521 | 0.000 | . | 0.027 | 0.080 | 0.266 | 0.947 | 0.000 | 0.599 | 0.058 |
| History of CVD  (no=1, yes=2), | Spearman’s rho | 0.074 | -0.048 | 0.222 | 0.004 | 0.025 | -0.052 | 1.000 | 0.139 | 0.007 | -0.007 | -0.001 | 0.057 | 0.129 |
|  | p-value | 0.002 | 0.040 | 0.000 | 0.872 | 0.278 | 0.027 | . | 0.000 | 0.772 | 0.768 | 0.965 | 0.015 | 0.000 |
| Hypertension  (no=1, yes=2), | Spearman’s rho | 0.099 | -0.041 | 0.421 | 0.202 | -0.075 | -0.041 | 0.139 | 1.000 | 0.041 | -0.042 | 0.059 | 0.079 | 0.136 |
|  | p-value | 0.000 | 0.081 | 0.000 | 0.000 | 0.001 | 0.080 | 0.000 | . | 0.082 | 0.074 | 0.011 | 0.001 | 0.000 |
| Hypertriglyceridemia (no=1, yes=2), | Spearman’s rho | 0.107 | -0.092 | -0.032 | 0.186 | 0.102 | 0.026 | 0.007 | 0.041 | 1.000 | 0.240 | 0.076 | 0.088 | 0.010 |
|  | p-value | 0.000 | 0.000 | 0.175 | 0.000 | 0.000 | 0.266 | 0.772 | 0.082 | . | 0.000 | 0.001 | 0.000 | 0.677 |
| Low HDL-cholesterolemia (no=1, yes=2), | Spearman’s rho | 0.112 | -0.115 | -0.003 | 0.123 | 0.096 | -0.002 | -0.007 | -0.042 | 0.240 | 1.000 | 0.024 | 0.057 | 0.048 |
|  | p-value | 0.000 | 0.000 | 0.902 | 0.000 | 0.000 | 0.947 | 0.768 | 0.074 | 0.000 | . | 0.306 | 0.015 | 0.040 |
| Hyper LDL cholesterolemia (no=1, yes=2), | Spearman’s rho | 0.076 | 0.149 | 0.093 | 0.136 | -0.105 | -0.172 | -0.001 | 0.059 | 0.076 | 0.024 | 1.000 | 0.033 | 0.043 |
|  | p-value | 0.001 | 0.000 | 0.000 | 0.000 | 0.000 | 0.000 | 0.965 | 0.011 | 0.001 | 0.306 | . | 0.158 | 0.066 |
| Diabetes  (no=1, yes=2), | Spearman’s rho | 0.107 | -0.070 | 0.055 | 0.088 | -0.005 | -0.012 | 0.057 | 0.079 | 0.088 | 0.057 | 0.033 | 1.000 | 0.048 |
|  | p-value | 0.000 | 0.003 | 0.020 | 0.000 | 0.839 | 0.599 | 0.015 | 0.001 | 0.000 | 0.015 | 0.158 | . | 0.042 |
| CKDepi  (no=1, yes=2), | Spearman’s rho | 0.084 | -0.028 | 0.237 | 0.060 | -0.019 | -0.044 | 0.129 | 0.136 | 0.010 | 0.048 | 0.043 | 0.048 | 1.000 |
|  | p-value | 0.000 | 0.232 | 0.000 | 0.010 | 0.416 | 0.058 | 0.000 | 0.000 | 0.677 | 0.040 | 0.066 | 0.042 | . |
